# Prevalence of Poor Mental Health Among Adolescents in Kabul, Afghanistan, as of November 2021

**DOI:** 10.1001/jamanetworkopen.2022.18981

**Published:** 2022-06-23

**Authors:** Sayed Jafar Ahmadi, Laura Jobson, Arul Earnest, Daniel McAvoy, Zeinab Musavi, Nasratullah Samim, Sayed Ali Akbar Sarwary

**Affiliations:** 1 Counseling Department, Kabul Education University, Kabul, Afghanistan; 2 Turner Institute for Brain and Mental Health, Monash University, Melbourne, Australia; 3 Department of Epidemiology and Preventive Medicine, School of Public Health and Preventive Medicine, Monash University, Melbourne, Australia; 4 Centre for Humanitarian Leadership, Deakin University, Melbourne, Australia; 5 Behrawan Research and Psychology Services Organization, Kabul, Afghanistan

## Abstract

This cross-sectional study assesses the mental health of adolescents in Afghanistan and evaluates their risk of having a psychiatric disorder by age and sex.

## Introduction

After 20 years of war with the US and its allies, the Taliban regained control of Afghanistan in August 2021. Afghanistan’s long history of conflict has affected the mental health of Afghan adolescents.^1,2^ In a sample of 1011 adolescents in Afghanistan, 224 (22.2%) met criteria for a probable psychiatric disorder and 182 (18.0%), 49 (4.8%), and 242 (23.9%) met criteria for emotional concerns, conduct problems, and posttraumatic stress disorder (PTSD), respectively.^2^ In the same study, female sex was associated with over twice the odds of having a probable psychiatric disorder.^2^ In another study, young individuals in Afghanistan were found to have poor mental health, and younger age was a risk factor.^3^ There have been rapid social and political changes in Afghanistan since these studies were conducted; therefore, the goal of the present study was to examine current mental health among adolescents in Afghanistan. We hypothesized that being female and of younger age would be associated with poorer mental health.

## Methods

The Afghan Ministry of Health Research Ethics Committee approved this study. Participants and parents or guardians provided written informed consent. The study followed the STROBE reporting guideline.

Participants were recruited from one of the largest high schools in Kabul and via community outreach. We invited 200 girls and 250 boys to participate. Data were collected between November 1 and December 31, 2021. Our primary outcome was poor mental health as indicated by scoring above cutoffs on the Child Revised Impact of Event Scale (≥30 [for PTSD]), the Mood and Feeling Questionnaire (short form (≥12 [for depression]), the Revised Children’s Manifest Anxiety Scale (≥19 [for anxiety]), and the Strengths and Difficulties Questionnaire (≥20); subscale cutoffs are provided in the Table and the eMethods in the Supplement. Analyses were conducted using IBM SPSS Statistics 26.0; hypothesis tests were 2 sided. Based on clinical cutoffs, we used binary outcomes and tested associations between outcomes and a priori risk factors (sex, age) using binary logistic regression analyses.

**Table.** Study Participant Characteristics by Sex and Age

Characteristic	Participants			OR (95% CI)	
	Boys (n = 216)	Girls (n = 160)	Total (n = 376)	Sex (female vs male)	Age (per-year increase)
**Demographic**					
Age, mean (SD), y	16.7 (2.0)	16.0 (2.0)	16.4 (2.0)	NA	NA
Education level, No. (%)^a^					
Grade 7	14 (6.5)	24 (15.0)	38	NA	NA
Grade 8	45 (20.8)	24 (15.0)	69		
Grade 9	22 (10.2)	22 (13.8)	44		
Grade 10	15 (6.9)	29 (18.1)	44		
Grade 11	78 (36.1)	29 (18.1)	107		
Grade 12	42 (19.4)	32 (20.0)	74		
**Mental health scoring**					
SDQ					
Mean (SD)	12.8 (5.6)	19.1 (5.2)	15.6 (6.2)	NA	NA
Clinical range (≥20), No. (% [95% CI])	30 (13.9 [10.1-19.8])	76 (47.5 [40.0-55.6])	106 (28.2 [24.5-34.1])	5.34 (3.25-8.77)	0.92 (0.83-1.03)
PTSD (CRIES)					
Mean (SD)	23.5 (13.0)	39.5 (11.6)	30.0 (14.8)	NA	NA
Clinical range (≥30), No. (% [95% CI])	67 (31.0 [25.0-37.0])	127 (79.4 [72.5-85.6])	194 (51.6 [46.3-56.4])	8.56 (5.30-13.82)	0.88 (0.79-0.97)
Depression (MFQ)					
Mean (SD)	8.18 (6.7)	16.54 (5.8)	11.99 (7.6)	NA	NA
Clinical range (≥12), No. (% [95% CI])	57 (26.4 [23.0-36.1])	127 (79.4 [73.1-85.6])	184 (48.9 [47.0-57.5])	9.05 (5.53-14.81)	0.88 (0.79-0.98)
Anxiety (RCMAS)					
Mean (SD)	13.0 (7.3)	21.4 (4.8)	16.9 (7.6)	NA	NA
Clinical range (≥19), No. (% [95% CI])	45 (20.8 [18.0-31.1])	125 (78.1 [71.9-83.8])	170 (45.2 [44.3-54.8])	10.95 (6.62-18.12)	0.86 (0.77-0.95)
**SDQ subscale scoring**					
Prosocial behavior					
Mean (SD)	6.77 (2.2)	6.32 (2.0)	6.6 (2.1)	NA	NA
Clinical cutoff ≤4, No. (% [95% CI])	32 (14.8 [10.6-20.8])	28 (17.5 [11.9-23.1])	60 (16.0 [12.5-19.9])	1.16 (0.67-2.02)	1.00 (0.87-1.14)
Emotional problems					
Mean (SD)	3.56 (2.7)	7.16 (2.3)	5.11 (3.1)	NA	NA
Clinical cutoff ≥7, No. (% [95% CI])	31 (14.4 [10.0-20.1])	103 (64.4 [56.9-71.7])	134 (35.6 [31.4-41.5])	10.38 (6.29-17.11)	0.87 (0.78-0.97)
Hyperactivity					
Mean (SD)	3.56 (2.09)	5.53 (2.20)	4.42 (2.35)	NA	NA
Clinical cutoff ≥8, No. (% [95% CI])	7 (3.2 [1.0-6.3])	33 (20.6 [14.4-26.9])	40 (10.6 [7.6-14.4])	7.42 (3.19-17.29)	0.89 (0.76-1.04)
Peer problems					
Mean (SD)	3.96 (1.6)	4.71 (1.6)	4.29 (1.7)	NA	NA
Clinical cutoff ≥5, No. (% [95% CI])	77 (35.6 [30.9-43.0])	86 (53.8 [46.3-61.9])	163 (43.4 [39.5-49.6])	1.96 (1.29-2.99)	0.97 (0.88-1.07)
Conduct problems					
Mean (SD)	3.29 (2.1)	4.58 (2.00)	3.85 (2.2)	NA	NA
Clinical cutoff ≥6, No. (% [95% CI])	34 (15.7 [11.1-21.7])	53 (33.1 [25.6-40.6])	87 (23.1 [19.6-28.3])	2.52 (1.54-4.13)	0.95 (0.85-1.07)

Abbreviations: CRIES, Child Revised Impact of Event Scale; MFQ, Mood and Feeling Questionnaire; NA, not applicable; OR, odds ratio; PTSD, posttraumatic stress disorder; SDQ, Strengths and Difficulties Questionnaire; RCMAS, Revised Children’s Manifest Anxiety Scale.

^a^ Percentages have been rounded and therefore may not total 100%.

## Results

Of the 450 adolescents invited to participate, 376 were included in the study (160 girls [42.6%], 216 boys [57.4%]); the mean (SD) age was 16.4 (2.0) years. Of these, 106 adolescents (28.2%) were at substantial risk for psychiatric problems and approximately half of participants met criteria for probable PTSD (194 [51.6%]), depression (184 [48.9%]), or anxiety (170 [45.2%]) (Table). Among girls, 47.5% were at substantial risk of having psychiatric problems (vs 13.9% of boys), and female sex was associated with a higher odds of having psychiatric problems, with more girls vs boys meeting criteria for probable diagnosis of PTSD (127 [79.4%] vs 67 [31.0%]), depression (127 [79.4%] vs 57 [26.4%]), and anxiety (125 [78.1%] vs 45 [20.8%]). Younger age was associated with probable psychiatric disorders (Table).

## Discussion

To our knowledge, this study is the first to assess mental health among adolescents in Afghanistan after the change in political leadership that occurred in Afghanistan in 2021. Nearly 29% of adolescents were at substantial risk of having psychiatric problems, and approximately half the sample met criteria for a probable diagnosis of PTSD, depression, or anxiety, rates higher than those observed previously.^2^ Female sex was associated with a higher odds of having a psychiatric disorder, and younger age was associated with poorer mental health.

This study has some limitations. Owing to heightened security, we were unable to adopt a random-sampling design, we could only recruit from Kabul, it was difficult to recruit girls, the sample size was limited, and the cross-sectional design precluded any inference of causation. Follow-up studies are needed to monitor the chronicity of psychiatric disorders among adolescents in Afghanistan.

Adolescents in Afghanistan, especially girls, are experiencing significant mental health problems. However, Afghanistan’s health system is struggling, and foreign humanitarian aid has diminished substantially.^1^ There is a need for mental health interventions that are tailored to the current political and social environment in Afghanistan. Furthermore, clinicians treating recently arrived adolescent refugees from Afghanistan must consider the emotional and behavioral presentations within the context of the political, historical, and social experiences of this population.

## Notes

Supplement.
eMethods.

eReferences
