# Supplementary material for: Prevalence of Poor Mental Health Among Adolescents in Kabul, Afghanistan, as of November 2021
Source: JAMA Netw Open. 2022 Jun 23;5(6):e2218981. doi: 10.1001/jamanetworkopen.2022.18981 (PMC9226996; doi:10.1001/jamanetworkopen.2022.18981)
Supplement: Supplement. — eMethods. eReferences [file jamanetwopen-e2218981-s001.pdf]

Supplemental Online Content

Ahmadi SJ, Jobson L, Earnest A, et al. Prevalence of poor mental health among adolescents in Kabul, Afghanistan, as of November 2021. *JAMA Netw Open*. 2022;5(6):e2218981. doi:10.1001/jamanetworkopen.2022.18981

eMethods.

eReferences

This supplemental material has been provided by the authors to give readers additional information about their work.

© 2022 Ahmadi SJ et al. *JAMA Network Open.*

eMethods

We report a cross-sectional study and STROBE guidelines were followed.

Participants

Eligibility criteria were: a) being an adolescent (10-24 years)[1]; b) living in Kabul; and c) able to complete the questionnaires in Dari/Pashto. Participants were recruited through a large local school and community connections. The local school is located in West Kabul and is a large public school that teaches both boys and girls in three shifts, with the first and third shift being for boys and the second shift being for girls. Our sample consisted of 376 (160 girls; 216 boys) general community members, aged 11-21 years (*M*=16.42, *SD*=2.02). All students were in Years 7-12. Students had on average 8.00 (*SD*=2.31) family members.

Measures

Child Revised Impact of Event Scale (CRIES [2])

The CRIES is a 13-item self-report measure of symptoms of PTSD[2]. It includes three subscales; intrusions, avoidance and arousal. The CRIES was self-completed by the adolescents. Adolescents responded to items on scales ranging from 0 (*not at all*) to 5 (*often*), with total scores ranging from 0 to 65 and higher scores indicating higher PTSD symptoms[2]. The suggested clinical cut-off score is 30[2]. The CRIES has been found to have good psychometric properties[2], including good convergent validity with the Spence Children's Anxiety Scale-20 and Short Mood and Feeling Questionnaire[3]. The CRIES has been used with Afghan adolescents[4][5]. In the current study internal consistency was good (Cronbach α=.86).

Strengths and Difficulties Questionnaire (SDQ[6])

The SDQ is a 25-item questionnaire that screens adolescents for positive (prosocial behaviour) and negative (emotional symptoms, conduct problems, hyperactivity/inattention,

© 2022 Ahmadi SJ et al. *JAMA Network Open.*

peer relationship problems) aspects over the past six months[6]. Five items refer to prosocial skills, and 20 items refer to difficulties. Each item is rated on a scale of “*not true*,” (0) “*somewhat true*,” (1) or “*certainly true*.”(2). In the current study the adolescents completed the self-report version. The total difficulty score is calculated by adding the results of the emotional, conduct, hyperactivity and peer problem subscales. Following previous research with Afghan youth[7], we used the cut-points for the 4-banded categorization of the SDQ scores to identify those adolescents that were “very high” risk; SDQ total scores of ≥20 were at substantial risk of clinically significant problems. Cut-off scores for the subscales were: Emotion ≥7, Conduct ≥6, Hyperactivity ≥8, Peer Problems ≥5, and Prosocial Behavior ≤4[8]. The SDQ has good psychometric properties, including convergent validity which been examined using the syndrome scales of the youth self-report version of the Child Behavior Checklist[9]. Internal consistency was good (Cronbach alphas >.77).

Mood and Feeling Questionnaire-Short Version (MFQ[10])

The MFQ which is a 13-item questionnaire based on criteria for depression. Each item was rated on a 3-point scale: "*true*", "*sometimes true*", and "*not true*" with respect to the events of the past two weeks. Items are summed to provide a total depression symptom, with scores ranging from 0 to 26 and higher scores indicating greater depression severity. A suggested clinical cut-off is ≥ 12[10]. The MFQ has good validity and reliability, including convergent validity – the MFQ is significantly correlated with the Spence Children's Anxiety Scale and the Pediatric Quality of Life Enjoyment and Satisfaction Questionnaire[11]. In the current study internal consistency was good (Cronbach α= .93).

Revised Children's Manifest Anxiety Scale (RCMAS[12])

The RCMAS-R was used to assess the level and nature of anxiety, as experienced by children, using a simple yes-or-no response format. It contains 37 items; 28 anxiety items and

© 2022 Ahmadi SJ et al. *JAMA Network Open.*

9 social desirability items. Scores on the anxiety items are totalled to give a total anxiety score, with higher scores indicating worse symptoms of anxiety. A total score of 19 (based on the 28 anxiety items) is considered clinically significant[12]. The reliability and validity of the RCMAS are good[12,13]. In the current study internal consistency was good (α=.92).

eReferences

[1] Sawyer, S.M., Azzopardi, P.S., Wickremarathne, D., & Patton, G.C. (2018). The age of adolescence. *The Lancet: Child and Adolescent Health, 2*, 223-228.

[2] Perrin, S., Meiser-Stedman, R., & Smith, P. (2005). The children's revised impact of event scale (CRIES): Validity as a screening instrument for PTSD. *Behavioural and Cognitive Psychotherapy, 33(4)*, 487–498. Doi: 10.1017/S1352465805002419

[3] Deeba, F., Rapee, R. M., & Prvan, T. (2014). Psychometric properties of the Children's Revised Impact of Events Scale (CRIES) with Bangladeshi children and adolescents. *PeerJ, 2*, e536. https://doi.org/10.7717/peerj.536

[4] Ahmadi, S. J., Kajbaf, M. B., Neshat Doost, H.T., Dalgleish, T., Jobson, L., & Mosavi, Z. (2018). The efficacy of memory specificity training in improving symptoms of post-traumatic stress disorder in bereaved Afghan adolescents. *Intervention;16*:243-8. Available from: https://www.interventionjournal.org/text.asp?2018/16/3/243/246434

[5] Panter-Brick, C., Eggerman, M., Gonzalez, V., & Safdar, S. (2009). Violence, suffering, and mental health in Afghanistan: a school-based survey. *Lancet (London, England), 374(9692)*, 807–816. https://doi.org/10.1016/S0140-6736(09)61080-1

[6] Goodman R, Meltzer H, Bailey V. (1998). The Strengths and Difficulties Questionnaire: a pilot study on the validity of the self-report version. *Eur Child Adolesc Psychiatry, 7(3):*125-30. doi: 10.1007/s007870050057. PMID: 9826298.

[7] Haar, K., El-Khani, A., Molgaard, V. et al. (2020). Strong families: a new family skills

© 2022 Ahmadi SJ et al. *JAMA Network Open.*

training programme for challenged and humanitarian settings: a single-arm intervention tested in Afghanistan. *BMC Public Health 20*, 634 (2020). https://doi.org/10.1186/s12889-020-08701-w

[8] Scoring the Strengths & Difficulties Questionnaire for age 4–172014. Available from: https://www.ehcap.co.uk/content/sites/ehcap/uploads/NewsDocuments/236/SDQEnglishUK4-17scoring-1.PDF.

[9] Vugteveen, J., de Bildt, A., Theunissen, M., Reijneveld, S. A., & Timmerman, M. (2021). Validity Aspects of the Strengths and Difficulties Questionnaire (SDQ) Adolescent Self-Report and Parent-Report Versions Among Dutch Adolescents. *Assessment, 28(2)*, 601–616. https://doi.org/10.1177/1073191119858416

[10] Angold, A., Costello, E. J., Messer, S. C., Pickles, A., Winder, F., & Silver, D. (1995). *Development of a short questionnaire for use in epidemiological studies of depression in children and adolescents* (Vol. 5, pp. 237–249).

[11] Thabrew, H., Stasiak, K., Bavin, L. M., Frampton, C., & Merry, S. (2018). Validation of the Mood and Feelings Questionnaire (MFQ) and Short Mood and Feelings Questionnaire (SMFQ) in New Zealand help-seeking adolescents. *International Journal of Methods in Psychiatric Research, 27(3),* 1–9.

[12] Reynolds, C.R., and Richmond, B.O. (1985). *Revised Children's Manifest Anxiety Scale. RCMAS Manual*. Los Angeles: Western Psychological Services.

[13] Stellard, P., Velleman, R., Langsford, J. & Baldwin, S. (2001). Coping and psychological distress in children involved in road traffic accidents", *British Journal of Clinical Psychology, 40*, 197-208.

© 2022 Ahmadi SJ et al. *JAMA Network Open.*
